# Diabetes: Chronic Metformin Treatment and Outcome Following Acute Stroke

**DOI:** 10.3389/fneur.2022.849607

**Published:** 2022-04-26

**Authors:** Naveed Akhtar, Rajvir Singh, Saadat Kamran, Blessy Babu, Shobana Sivasankaran, Sujatha Joseph, Deborah Morgan, Ashfaq Shuaib

**Affiliations:** ^1^The Neuroscience Institute, Hamad Medical Corporation, Doha, Qatar; ^2^Cardiology Research Center, Hamad Medical Corporation, Doha, Qatar; ^3^Neurology Division, Department of Medicine, University of Alberta, Edmonton, AB, Canada

**Keywords:** ischemic stroke, diabetes, metformin, outcome, mortality

## Abstract

**Aim:**

To evaluate if in patients with known diabetes, pretreatment metformin will lead to less severe stroke, better outcome, and lower mortality following acute stroke.

**Methods:**

The Qatar stroke database was interrogated for stroke severity and outcome in patients with ischemic stroke. Outcome was compared in nondiabetic vs. diabetic patients and in diabetic patients on metformin vs. other hypoglycemic agents. The National Institute of Health Stroke Scale was used to measure stroke severity and 90-day modified Rankin scale (mRS) score to determine outcome following acute stroke.

**Results:**

In total, 4,897 acute stroke patients [nondiabetic: 2,740 (56%) and diabetic: 2,157 (44%)] were evaluated. There were no significant differences in age, risk factors, stroke severity and type, or thrombolysis between the two groups. At 90 days, mRS (shift analysis) showed significantly poor outcome in diabetic patients (*p* < 0.001) but no differences in mortality. In the diabetic group, 1,132 patients were on metformin and 1,025 on other hypoglycemic agents. mRS shift analysis showed a significantly better outcome in metformin-treated patients (*p* < 0.001) and lower mortality (8.1 vs. 4.6% *p* < 0.001). Multivariate negative binomial analyses showed that the presence of diabetes negatively affected the outcome (90-day mRS) by factor 0.17 (incidence risk ratio, IRR, 1.17; CI [1.08–1.26]; *p* < 0.001) when all independent variables were held constant. In diabetic patients, pre-stroke treatment with metformin improved the outcome (90-day mRS) by factor 0.14 (IRR 0.86 [CI 0.75–0.97] *p* = 0.006).

**Conclusion:**

Similar to previous reports, our study shows that diabetes adversely affects stroke outcome. The use of prior metformin is associated with better outcome in patients with ischemic stroke and results in lower mortality. The positive effects of metformin require further research to better understand its mechanism.

## Introduction

Metformin is a first-line oral hypoglycemic agent used to treat type 2 diabetes (1). It works by reducing intestinal glucose absorption and increases hepatic and muscle glucose uptake (1, 2). Metformin enhances energy metabolism, reduces oxidative stress, and inhibits proteostasis, leading to improved balance of survival and death signaling in multiple cell types, including neurons (3). These effects, especially the antioxidant activity, may be because of the AMP-activated protein kinase (AMPK), an enzyme with important regulatory functions in energy regulation (4, 5). Additionally, metformin may provide protective effects on the integrity of the neurovascular unit by improving function of endothelial cells, pericytes, astrocytes, and the blood–brain barrier integrity (6). It is therefore not surprising that there has been considerable interest in the use of metformin as an experimental neuroprotective agent (7, 8), and clinical reports suggest neuroprotection in patients with Type 2 diabetes who suffer acute ischemic stroke (9, 10).

Diabetes mellitus (DM) is an important risk factor for stroke and its incidence is increasing. We have previously shown that prediabetes and DM were present in ~70% of patients presenting with acute stroke in Qatar and it was associated with higher rates of small vessel disease and early stroke recurrence (11, 12). We have also previously shown that the younger age at onset of symptoms and higher rates of patients with milder disease were also likely related to the poorly controlled hypertension and diabetes mainly in this Southeast Asian population (11, 12). A recent meta-analysis of 66 studies with predominantly Caucasian population revealed that the presence of DM was however associated with more severe symptoms, increased risk of medical complications, prolonged hospitalization, higher mortality, and increased rates of readmissions (13). DM also increased the risk of complications with intravenous recombinant tissue plasminogen activator (rt-PA) in acute stroke, resulting in a less favorable clinical outcome (14). A recent intriguing report from Europe suggests that diabetic patients on chronic treatment with metformin have less severe stroke and improved outcome in patients treated with rt-PA (15).

These data on the usefulness of metformin in acute stroke, while promising, have limitations. Earlier studies had few patients and did not compare outcome in diabetic patients to nondiabetic patients (9, 10). Similarly, in the larger recent study on the benefits of metformin in patients treated with intravenous rt-PA, the comparison was also lacking due to the unavailability of a nondiabetic group (15). To better understand the potential neuroprotection with metformin, we explored its effects in a large database from Qatar where diabetes is common, and a large portion of the patients are on metformin for type 2 DM (11, 12). There were three main objectives for the current study: (1) to evaluate whether the presence of prior DM leads to more severe stroke, slows recovery, and adversely affects the prognosis in acute stroke; (2) to evaluate whether diabetic patients on metformin present with milder symptoms in acute stroke; and (3) to evaluate whether metformin use in diabetes was associated with a better recovery and improved prognosis.

## Methods

We collected clinical details on all patients with acute stroke admitted to the Hamad General Hospital (HGH) and prospectively entered into the Qatar Stroke database. The registry was established in 2013 and now has data on more than 11,000 patients. The details of the registry have previously been published (11, 12). In brief, HGH is a Joint Commission International-accredited 600-bed hospital, and ~95% of suspected strokes (~2,500/year) for the state of Qatar are admitted here. The Institutional Review Board of Hamad Medical Corporation at the Medical Research Center approved the study (MRC-15304/15). The patient data were collected prospectively and entered into the database by qualified nurse specialists. Patient identification data were deleted and there was no consent for individual patients when the data were analyzed retrospectively.

### Data Availability

All data relevant to the study are included in the article or uploaded as supplementary information, and data are available upon reasonable request.

### Patient and Public Involvement

Patients or the public *were not* involved in the design, conduct, reporting, or dissemination plans of our research.

### Patient Characteristics

Patient characteristics including age, sex, nationality, medical comorbidities, and prior medication were collected into the HGH Stroke registry. Data from emergency medical services (EMS), immediate emergency department (ED) care, door-to-needle time (for thrombolysis patients), severity of symptoms as measured by the National Institute of Health Stroke Scale score (NIHSS score), length of stay (LOS) in the hospital, timing and completion of investigations, neuroimaging, post-stroke complications, and in-hospital mortality were recorded for all patients. The stroke etiology was recorded according to the TOAST (Trial of Org10172 in Acute Stroke Treatment) criteria as previously described (11, 12). The modified Rankin scale (mRS) measurements were done at discharge and at 90 days following the onset of symptoms. We used the 90-day mRS scores for our primary outcome analysis. Both dichotomous (good: mRS of ≤ 0–2 or poor: mRS of 3–6) and shift analyses were used to evaluate the outcome. Shift analysis of ordinal scores was the most efficient techniques to capture treatment effects (16, 17).

DM was diagnosed according to the American Diabetes Association (ADA) and WHO recommendations as previously report (11, 12), and included patients with a previous diagnosis of DM, on medication for DM or a HbA1c ≥6.5%, and the diagnosis of pre-DM was based on a HbA1c of 5.7–6.4% as per 2015 ADA clinical practice recommendations.

### Patient Disposition

Patients were admitted to the stroke ward and received acute care by a multidisciplinary team that was composed of stroke neurologists, clinical nurse specialists, stroke-trained physical, occupational, speech, and language therapists, and acute rehabilitation physicians. The stroke protocol began immediately upon admission and specific attention was paid to preventive measures to minimize the risk of aspiration pneumonia, bladder infection, venous thrombosis, and pressure ulcers.

We analyzed the clinical course and prognosis according to the following groups. The patients were subdivided into three groups: Group 1—subjects with stroke but no history of DM; Group 2—DM patients not on metformin; and Group 3—known DM patients on chronic metformin treatment. The mechanism of stroke, severity at presentation, risk of complications, LOS in hospital, and the prognosis at discharge and at 90 days were assessed in relation to the diagnosis of prediabetes and DM.

### Data Analysis and Statistics

Descriptive results for all quantitative variables (e.g., age) were reported as mean ± standard deviation (SD). Numbers (percentage) were reported for all qualitative variables (e.g., gender). The distribution of continuous variables was assessed prior to using statistical tools.

Independent sample *t*-tests were used to compare the average for all the quantitative variables between patients with and without diabetes. Pearson's chi-squared tests were used to compare the proportion of all qualitative variables between patients with and without diabetes.

The dependent variable mRS 90 at 90 days was discrete variable and each subject was having same length of observation time. The variance of the variable was more than two times of the mean. Assuming that variable was overdispersed and did not have zeros, we used multivariate negative binomial regression analysis to estimate expected rate of change in the dependent variable on behalf of independent variables in the study. Incidence risk ratios (IRRs) and their 95% confidence interval (CI) with values of *p* were presented in the tables. Separate negative binomial regression analyses were performed in patients with ischemic stroke with and without diabetes and in diabetic patients between patients on metformin vs. other hypoglycemic agents. A “*p*” value of 0.05 (two-tailed) was considered significant. SPSS 26·0 statistical package (IBM, Chicago, IL, United States) was used for the analysis.

## Results

### Study Population and Baseline Characteristics

There were 11,063 patients admitted to the HGH between 2013 and 2020 with a suspected diagnosis of acute stroke. After excluding patients with stroke mimics (3,138), Transient Ischemic Attacks (TIAs) (1,166), Intracerebral Hemorrhage (ICH) (1,235), and cerebral venous thrombosis (CVT; 126), 5,399 patients with ischemic stroke were available for analysis. As previous experimental research suggests that only chronic metformin treatment has neuroprotective effects, we also excluded patients in whom the diagnosis of DM (new onset diabetes) was made during admission and treatment initiated following the acute stroke (new onset diabetes: 501). There were 4,897 patients available for further analysis for this study and were reviewed in the following groups: acute stroke and no history of diabetes 2,740 (56%) and 2,157 (44%) with previous history of diabetes. Hyperglycemia (glucose levels of more than 10 mmol on initial reading) was seen in 880 of 2,740 patients with no diabetes. This improved to normal levels during the hospitalization.

**Figure 1:**
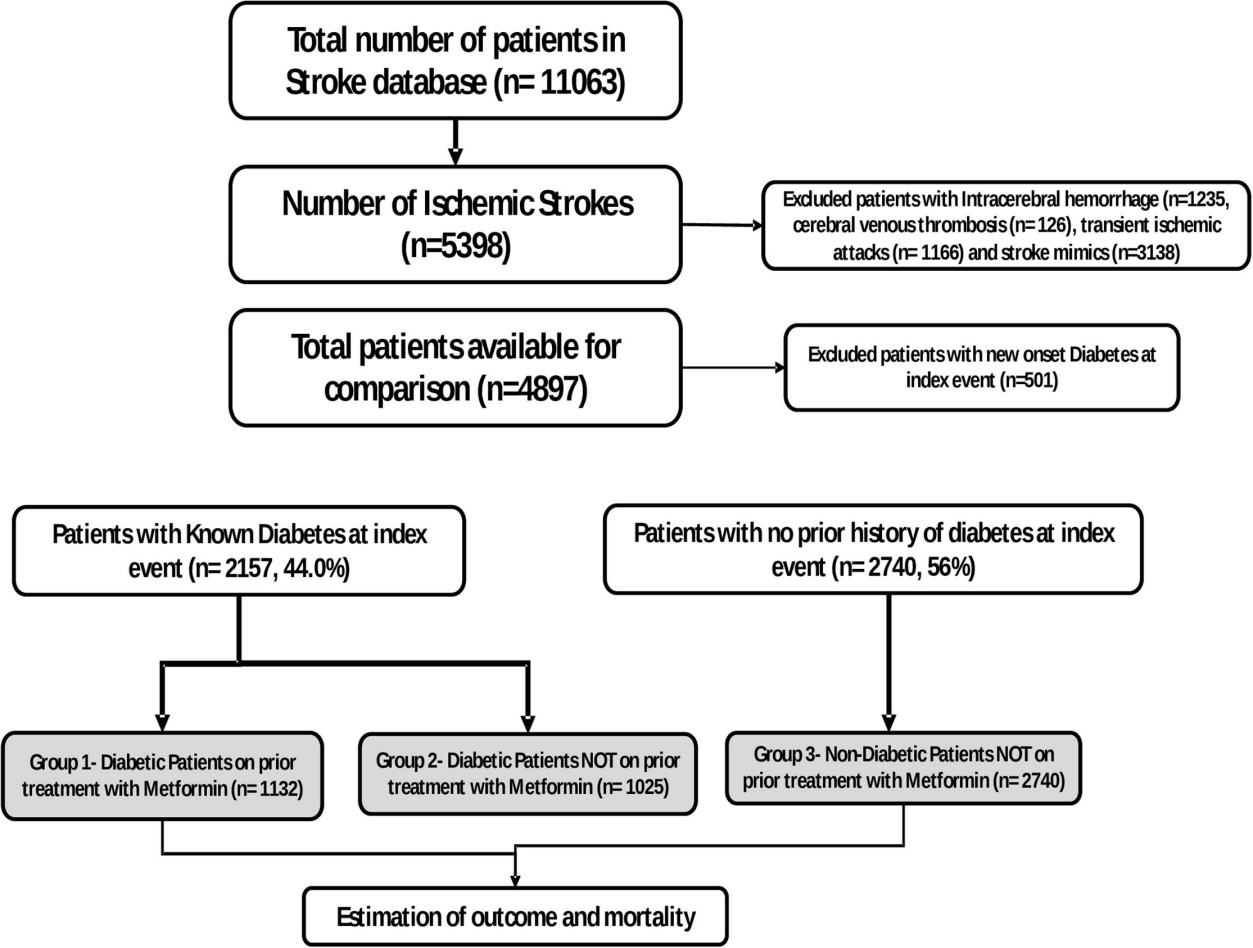
Flowcharts of the patient included in the study.

The 90-day mRS score was similar in patients with or with stroke onset hyperglycemia (mRS of 0–2 in 69.9% of patients with and without hyperglycemia). At 90-day follow-up, 728 (14.8%) patients were not available for evaluation. Most of the patients were expatriates and had likely left the country following their stroke. There were 279 deaths of patients (6.7%) during the 90-day follow-up. Unfortunately, we do not have data on the causes of mortality for most patients. The diabetic group comprised 1,025 patients with diabetes but not on metformin and 1,132 patients on metformin prior to stroke onset (see Figure 1 for details). The medications in the “non-metformin” group included gliclazide, glibenclamide, glimepiride, sitagliptin, vildagliptin, and dapagliflozin. During hospitalization and at discharge, 872 patients were taking insulin. There was no significant difference in the number of patients taking insulin in the two groups. The effects of insulin on outcome were not analyzed independently.

There were no significant differences in age, vascular risk factors, or the percentage of patients with a previous history of heart disease or stroke between patients in the three groups. Similarly, patients taking medications for hypertension or high cholesterol were equal in the two groups as was the percentage of patients on antithrombotic agents. The serum creatinine levels were similar in the two groups (metformin group: 95.8 ± 64.4; no metformin group: 97.0 ± 66.6; *p* = 0.57). The HbA1c was also similar between the diabetic patients on metformin and not on metformin. The details are shown in Tables 1–3.

**Table 1:** Comparison and outcome of Ischemic Strokes in Diabetics vs. Nondiabetics.

**Characteristic or Investigations**	**Total (*n* = 4,897)**	**Non-Diabetics (*n* = 2,740, 56.0%)**	**Diabetics (*n* = 2,157, 44.0%)**	***P*-value**
Age, Mean, years	54.7 ± 13.2	54.8 ± 13.2	54.5 ± 13.1	0.30
Men (%)	3,975 (81.2)	2,223 (81.1)	1,752 (81.2)	0.93
Hypertension	3,668 (74.9)	2,054 (75.0)	1,614 (74.8)	0.91
Dyslipidemia	2,618 (53.3)	1,458 (53.2)	1,160 (53.8)	0.69
Coronary artery disease	545 (11.1)	302 (11.0)	243 (11.3)	0.79
Atrial fibrillation	233 (4.8)	143 (5.2)	90 (4.2)	0.09
Active smoking	1,441 (29.4)	815 (29.7)	626 (29.0)	0.58
History of stroke	589 (12.0)	318 (11.6)	271 (12.6)	0.31
Obesity (BMI ≥ 30 kg/m^2^ (*n* = 4,697)	1,261 (26.8)	714 (27.2)	547 (26.4)	0.52
History of CKD	395 (8.1)	226 (8.2)	169 (7.8)	0.59
HbA1c % (*n* = 4746)	7.5 ± 2.9	7.4 ± 2.4	7.6 ± 3.5	0.03
NIHSS on admission	5.2 ± 5.5	5.4 ± 5.6	5.1 ± 5.4	0.08
NIHHS severity				
Mild (mRS 0–4)	3,031 (61.9)	1,659 (60.5)	1,372 (63.6)	0.09
Moderate (mRS 5–10)	1,162 (23.7)	670 (24.5)	492 (22.8)	
Severe (mRS > 10)	704 (14.4)	411 (15.0)	293 (13.6)	
Thrombolysis given	620 (12.7)	362 (13.2)	258 (12.0)	0.19
Post t-PA ICH (*n* = 500)	29 (5.8)	15 (4.8)	14 (7.4)	0.23
Thrombectomy done	229 (4.7)	127 (4.6)	102 (4.7)	0.88
Admitted under ICU care	129 (2.6)	75 (2.7)	54 (2.5)	0.61
Door to needle time (*n* = 620)	59.7 ± 41.4	59.6 ± 44.1	59.9 ± 37.4	0.94
Length of stay	5.9 ± 11.5	6.1 ± 12.7	5.6 ± 9.8	0.14
Modified Rankin Score- at discharge				
0	1,019 (20.8)	612 (22.3)	407 (18.9)	0.001
1	921 (18.8)	541 (19.7)	380 (17.6)	
2	814 (16.6)	442 (16.1)	372 (17.2)	
3	699 (14.3)	340 (12.4)	359 (16.6)	
4	834 (17.0)	464 (16.9)	370 (17.2)	
5	440 (9.0)	231 (8.4)	209 (9.7)	
6	170 (3.5)	110 (4.0)	60 (2.8)	
Modified Rankin Score- At 90 days (4,169)				
0	1,677 (40.2)	989 (43.7)	688 (36.1)	<0.001
1	738 (17.7)	404 (17.9)	334 (17.5)	
2	386 (9.3)	189 (8.4)	197 (10.3)	
3	465 (11.2)	227 (10.0)	238 (12.5)	
4	341 (8.2)	179 (7.9)	162 (8.5)	
5	283 (6.8)	115 (5.1)	168 (8.8)	
6	279 (6.7)	160 (7.1)	119 (6.2)	
Mortality – at discharge	170 (3.5)	110 (4.0)	60 (2.8)	0.02
Mortality at 90-days (*n* = 4,169)	279 (6.7)	160 (7.1)	119 (6.2)	0.29
TOAST classification				
Small vessel disease	2,428 (49.6)	1,360 (49.6)	1,068 (49.5)	0.78
Large vessel disease	1,052 (21.5)	589 (21.5)	463 (21.5)	
Cardio embolic stroke	693 (14.2)	399 (14.6)	294 (13.6)	
Stroke of determined origin	498 (10.2)	271 (9.9)	227 (10.5)	
Stroke of undetermined origin	226 (4.6)	121 (4.4)	105 (4.9)	

*BMI, body mass index; CKD, chronic kidney disease; HbA1c, hemoglobin A1c, NIHSS National Institutes of Health Stroke Scale, mRS, modified Rankin Score; ICU, Intensive Care Unit, ICH, Intracerebral hemorrhage*.

**Table 2:** Comparison and outcome of Ischemic Strokes in Diabetics with or without Prior Metformin treatment.

**Characteristic or Investigations**	**Total (*n* = 2,157)**	**Diabetics NOT on Prior Metformin** **(*n* = 1,025, 47.5%)**	**Diabetics on Prior Metformin** **(*n* = 1,132, 52.5%)**	***P*-value**
Age, Mean, years	54.4 ± 13.1	54.6 ± 13.1	54.4 ± 13.2	0.72
Men (%)	1,752 (81.2)	842 (82.1)	910 (80.4)	0.29
Hypertension	1,614 (74.8)	766 (74.7)	848 (74.9)	0.92
Dyslipidemia	1,160 (53.8)	537 (52.4)	623 (55.0)	0.22
Coronary Artery Disease	243 (11.3)	112 (10.9)	131 (11.6)	0.64
Atrial Fibrillation on Admission	90 (4.2)	42 (4.1)	48 (4.2)	0.87
Active Smoking	626 (29.0)	276 (26.9)	350 (30.9)	0.04
History of Stroke	271 (12.6)	137 (13.4)	134 (11.8)	0.28
Obesity (BMI ≥ 30 kg/m^2^ (*n* = 2,074)	547 (26.4)	258 (26.5)	289 (26.3)	0.93
History of CKD	169 (7.8)	83 (8.1)	86 (7.6)	0.67
HbA1c % (*n* = 5,213)	7.6 ± 3.5	7.6 ± 4.3	7.5 ± 2.5	0.49
NIHSS on admission	5.1 ± 5.4	5.0 ± 5.2	5.2 ± 5.7	0.54
NIHHS Severity				
Mild (mRS 0–4)	1,372 (63.6)	649 (63.3)	723 (63.9)	0.79
Moderate (mRS 5–10)	492 (22.8)	240 (23.4)	252 (22.3)	
Severe (mRS > 10)	293 (13.6)	136 (13.3)	157 (13.9)	
Thrombolysis Given	258 (12.0)	119 (11.6)	139 (12.3)	0.63
Post t-PA ICH (*n* = 189)	14 (7.4)	5 (6.4)	9 (8.1)	0.66
Thrombectomy Done	102 (4.7)	44 (4.3)	58 (5.1)	0.36
Admitted under ICU care	54 (2.5)	28 (2.7)	26 (2.3)	0.52
Door to Needle Time (*n* = 690)	59.8 ± 37.4	59.8 ± 39.8	59.9 ± 35.4	0.99
Length of Stay	5.6 ± 9.8	5.8 ± 12.6	5.5 ± 6.2	0.48
NIHSS At Discharge	3.9 ± 5.1	4.2 ± 5.1	3.8 ± 5.1	0.05
Modified Rankin Score- At Discharge				
0	407 (18.9)	169 (16.5)	238 (21.0)	0.08
1	380 (17.6)	185 (18.0)	195 (17.2)	
2	372 (17.2)	178 (17.4)	194 (17.1)	
3	359 (16.6)	164 (16.0)	195 (17.2)	
4	370 (17.2)	195 (19.0)	175 (15.5)	
5	209 (9.7)	103 (10.0)	106 (9.4)	
6	60 (2.8)	31 (3.0)	29 (2.6)	
Modified Rankin Score- 90 Days (*n* = 1,906)				
0	688 (36.1)	290 (32.8)	398 (38.9)	<0.001
1	334 (17.5)	156 (17.6)	178 (17.4)	
2	197 (10.3)	104 (11.8)	93 (9.1)	
3	238 (12.5)	97 (11.0)	141 (13.8)	
4	162 (8.5)	79 (8.9)	83 (8.1)	
5	168 (8.8)	86 (9.7)	82 (8.0)	
6	119 (6.2)	72 (8.1)	47 (4.6)	
Mortality at 90-Days (*n* = 4,589)	119 (6.2)	72 (8.1)	47 (4.6)	0.001

*BMI, body mass index; CKD, chronic kidney disease; HbA1c, hemoglobin A1c; NIHSS, National Institutes of Health Stroke Scale; mRS, modified Rankin Score; ICU, Intensive Care Unit; ICH, Intracerebral hemorrhage*.

**Table 3:** Multivariate negative binomial regression analysis for MRS at 90-days in patients with diabetes vs. non-diabetic patients.

**Characteristic**	**Odds ratio**	**95.0% CI**		***P*-value**
		**Lower**	**Upper CI**	
Age	1.001	0.99	1.004	0.67
Female	0.98	0.88	1.09	0.65
DM	1.17	1.08	1.26	0.001
NIHSS on Admission	1.004	0.99	1.01	0.29
Hypertension	0.94	0.85	1.04	0.15
Prior Stroke	1.17	1.001	1.27	0.02
Coronary Artery Disease	0.96	0.83	1.09	0.41
AF on Admission	0.99	0.82	1.21	0.95
Thrombolysis Given	1.00	0.88	1.15	0.92
Any Complication during admission	1.11	0.74	1.31	0.10
Intercept	1.87	1.28	2.75	0.001

*DM, Diabetes; NIHSS, National Institute of Health Stroke Scale; AF, Atrial fibrillation; BP, Blood Pressure*.

The clinical presentations, including the types of stroke (TOAST classification) and severity of symptoms, were similar in the three groups (as shown in Table 1). The majority of patients had mild stroke (mean NIHSS score 5.3 ± 5.6). This is similar to previous reports from Qatar (11, 12), likely related to the high incidence of uncontrolled hypertension and diabetes in the expatriate population. Patients with small vessel disease comprised 49% of patients and were similar in the three groups. The severity of stroke as measured on the NIHSS was similar in the three groups. The number of patients with mild stroke (NIHSS score <5), moderate stroke (NIHSS score 5–10), and severe stroke (NIHSS score >10) was similar in the three groups. The patients with severe stroke were however marginally more frequent in patients with no diabetes (15 vs. 13.6%) as shown in Table 1.

As previous work has suggested that patients on metformin had better outcome when treated with intravenous rt-PA (15), we evaluated the effects of treatment with rt-PA in our subjects. In the current study, there were no differences in the 90-day outcome as measured with mRS in patients treated rt-PA or thrombectomy in the three groups. The outcomes as measured with mRS at discharge and at 90-day follow-up were also similar in the three groups. The rates of rt-PA-related ICH were also similar in the three groups as were the rates of medical complications. Marginally, fewer patients on metformin were admitted to ICU, and the LOS in the hospital was also marginally less in patients taking metformin (see Tables 1, 2 for details).

### Outcome Analysis

**Figure 2:**
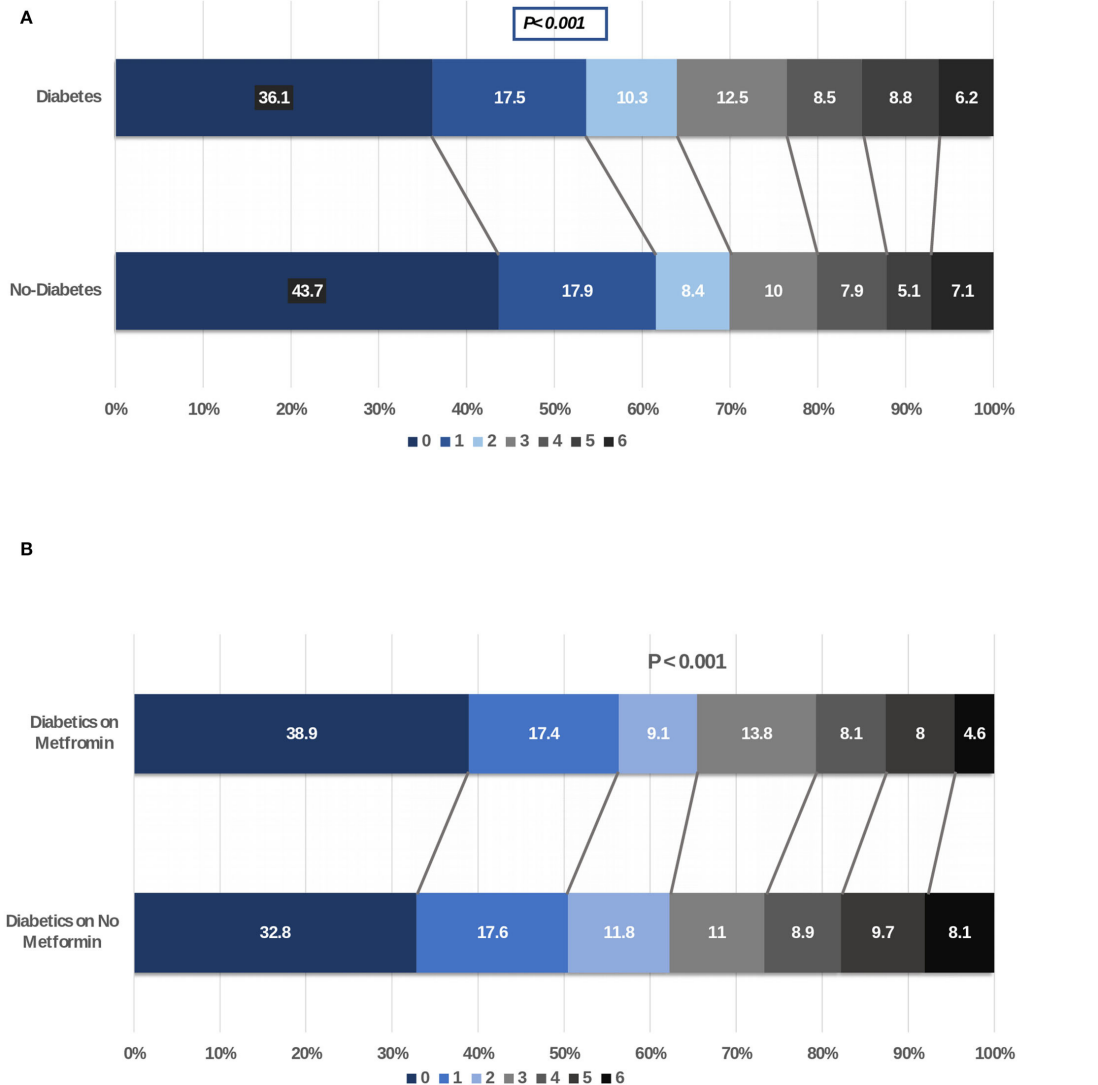
**(A)** Shift analysis comparing modified Rankin scale score at 90 days of patients with ischemic stroke with and without diabetes. **(B)** Shift analysis comparing modified Rankin scale score at 90 days of patients with ischemic stroke with diabetes on metformin vs. other hypoglycemic agents.

For our first objective, we compared the stroke severity (as measured on NIHSS), stroke subtypes, duration of hospitalization, recovery at discharge and at 90 days (as measured with mRS), and mortality in patients with stroke without and with a history of diabetes. Recovery was slower in diabetic patients at discharge and at 90 days and fewer patients had mRS of 0–2 at discharge (53.7 vs. 58.2%; *p* = 0.002) and at 90 days (64 vs. 69.9%; *p* < 0.001). In addition, while the mortality was significantly higher in patients without diabetes at discharge (4.0 vs. 2.4%; *p* = 0.02), this was not evident at 90 days (6.2 vs. 7.1%; *p* = 0.29). Shift analysis of the mRS showed a significantly lower score in patients with diabetes at discharge and at 90-day follow-up as shown in Figure 2A.

**Figure 3:**
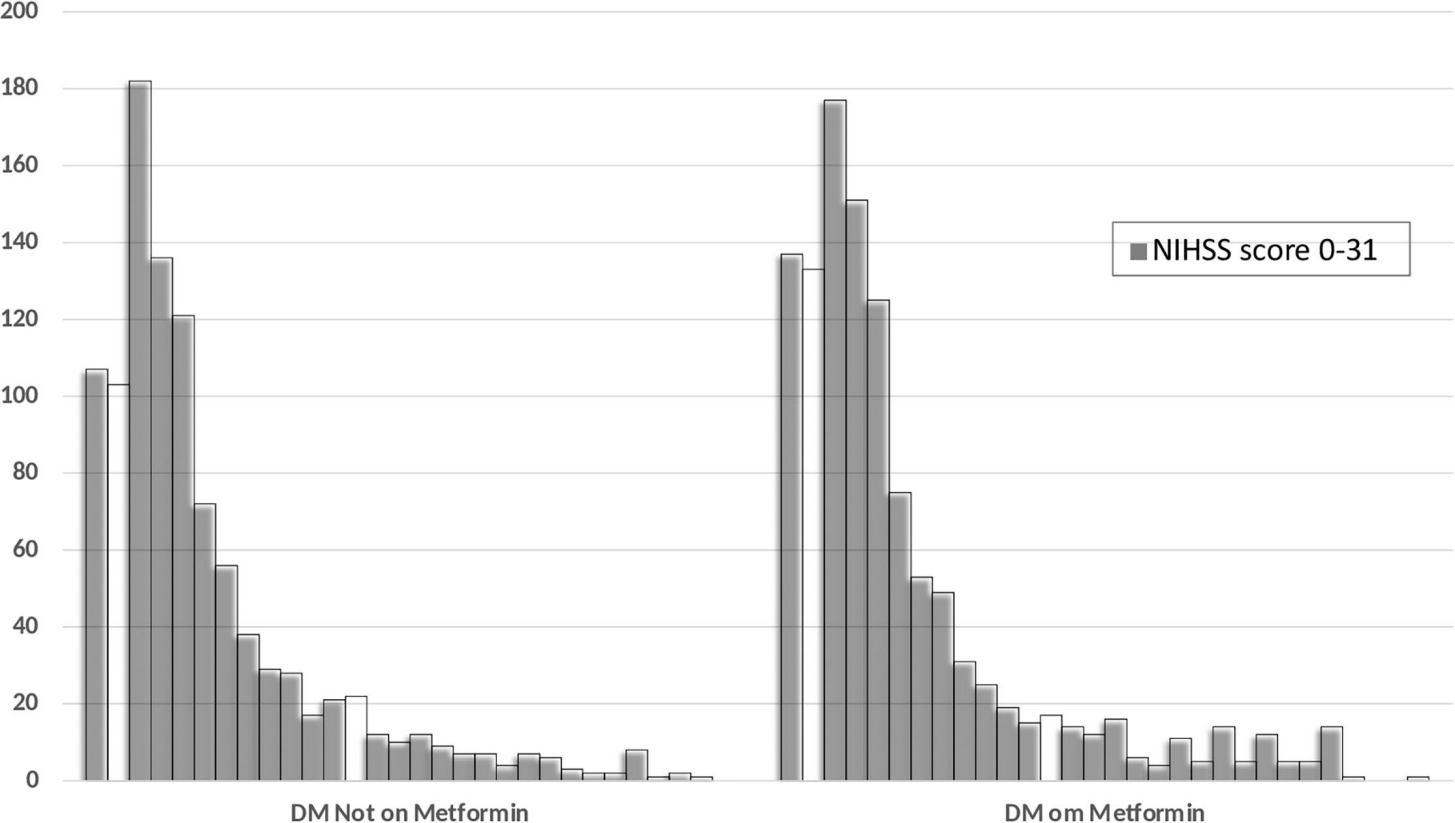
Distribution of the NIHSS score at admission among the diabetic patients with and without prior metformin therapy.

For our second objective, we did not identify any differences in the severity of stroke as measured on NIHSS between patients with diabetes (with or without metformin) or without diabetes. There were also no differences in the percentage of patients with mild, moderate, or severe severity of symptoms in the three groups. The severity of stroke symptoms as measured on the NIHSS between the diabetic patients with and without metformin use is shown in Figure 3.

For our final objective, we compared functional outcomes as measured with the mRS at discharge and at 90-day follow-up in diabetic patients with acute stroke. Our goal was to compare if chronic treatment with metformin will improve the outcome and prognosis. In our cohort of 2,157 patients with diabetes and acute stroke, 47.5% of patients were not on metformin. There were marginal differences in better outcome at discharge and at 90 days in metformin-treated patients when outcome was measured with mRS dichotomized at 0–2 vs. 3–6 (discharge: 55.4 vs. 51.9; *p* = 0.10; 90-day follow-up: 65.5 vs. 62.2%; *p* = 0.14) in metformin-treated patients. Mortality at 90-day follow-up was significantly lower in diabetic patients on metformin compared to patients on other oral hypoglycemic agents (4.6 vs. 8.1%; *p* = 0.001). Shift analysis of the mRS score showed a significantly better outcome in the metformin-treated patients at discharge and at 90-day follow-up as shown in Figure 2B.

### Multivariate Analysis

The details are shown in Tables 3, 4. There were significant differences in outcome in diabetic vs. nondiabetic patients with diabetic patients likely to have a worse outcome in the 90-day mRS shift analysis. This is similar to previous reports showing that the presence of diabetes adversely affects outcome (13). We next compared the 90-day outcome in patients with diabetes and acute stroke between patients on metformin and patients on other hypoglycemic agents. Diabetic patients on metformin were significantly more likely to have a better outcome on the 90-day shift analysis compared to patients on other hypoglycemic agents.

**Table 4:** Multivariate negative binomial regression analysis for MRS at 90-days in diabetic ischemic stroke patients on prior metformin vs. no-metformin.

**Characteristic**	**Odds ratio**	**95.0% CI**		***P*-value**
		**Lower**	**Upper CI**	
Age	1.0	0.99	1.01	0.94
Female	0.98	0.84	1.15	0.77
Metformin	0.86	0.75	0.97	0.006
NIHSS on Admission	1.01	0.99	1.02	0.14
Hypertension	0.89	0.73	1.10	0.17
Prior Stroke	1.12	0.95	1.34	0.07
Smoking	0.96	0.84	1.09	0.45
Coronary Artery Disease	1.08	0.89	1.30	0.33
AF on Admission	0.92	0.67	1.26	0.52
Thrombolysis Given	0.87	0.72	1.05	0.08
Any Complication during admission	1.12	0.87	1.144	0.24
Intercept	1.85	0.86	4.0	0.06

*DM, Diabetes; NIHSS, National Institute of Health Stroke Scale; AF, Atrial fibrillation; BP, Blood Pressure*.

Given that all other independent variables were held constant, presence of DM negatively affected outcome on mRS at 90 days by factor 0.17 (IRR 1.17 [CI 1.08–1.26] *p* = 0.001). In diabetic patients, pre-stroke treatment with metformin improved outcome on mRS at 90 days by factor 0.14 (IRR 0.86 [CI: 0 75–0.97] *p* = 0.006) when all other independent factors were held constant in the models (see Table 4 for details).

## Discussion

This is the largest study evaluating the potential neuroprotective effects of metformin in diabetic patients with acute stroke. Similar to our previous studies from Qatar, the neurological symptoms were mild in most patients. This is likely related to the high incidence of untreated hypertension and diabetes in our cohort of relatively young males resulting in more frequent subcortical lacunar strokes (11, 12). Our study showed that patients with diabetes were less likely to have a good outcome when compared to nondiabetic patients. Unlike the observations from previous studies where patients on metformin had less severe stroke symptoms (10, 15), in our study, the severity of symptoms at admission appeared to be similar in diabetic patients on metformin or on other hypoglycemic agents. Our study however confirms that diabetic patients taking metformin at the time of their stroke were more likely to have improved outcome when compared to diabetic patients not on metformin. The mortality rates were also significantly lower in metformin-treated patients at discharge and at 90-day follow-up. The higher mortality in the nondiabetic group at the time of discharge is difficult to explain, but at 90-day follow-up, there were no differences in the mortality in the two groups.

Similar to our research, several previous studies have shown that diabetes adversely affects stroke outcome (13, 18–23). Patients with diabetic stroke are more likely to have complications, with longer hospitalizations and a poor outcome. There is considerable debate whether the harmful effects are directly related to diabetes or secondary to the hyperglycemia commonly evident in such patients. Hyperglycemia during an acute stroke is more common in large ischemic stroke. It has been known for some time that this so-called “stress-hyperglycemia” is associated with a poor prognosis (18). It is therefore important to measure the HbA1c, which is more reflective of the steady state glucose in the days prior to the stroke. Higher HbA1c are associated with severity of symptoms and worse outcomes (13, 19). In addition, the presence of diabetes leads to higher risk of stroke recurrence (21, 22) and increased mortality (23).

Four previous reports have documented the positive effects of metformin in stroke patients (9, 10, 15, 24). The study by Mima et al. (10) evaluated acute stroke in a small study of 335 patients. Patients on metformin had milder symptoms (more patients with NIHSS score of <3) and had better outcome. Similarly, the study from Iran showed fewer deaths in metformin-treated stroke patients during follow-up when compared to glyburide-treated patients with type 2 diabetes (9). In a larger multicenter study from Europe, 1,919 patients with diabetes who underwent thrombolysis for acute stroke were evaluated (15). Thirty-nine percent of patients were on metformin treatment. Patients on metformin had lower NIHSS scores on admission, higher number of patients functionally independent at 3 months, and significantly lower mortality (12 vs. 18%) compared to patients not taking metformin. A possible shortcoming of the study is the lack of comparison between the patient groups with diabetic and nondiabetic strokes (15). The authors postulated that metformin may have neuroprotective effects and therefore the milder strokes. In a survey of patients with acute stroke and diabetes from Denmark, reported in 2012, the use of metformin was associated with lower mortality that was not evident in patients taking sulphonylureas (24). Our study comprehensively compared the clinical features and outcome between patients with acute stroke with or without diabetes and in diabetic patients on chronic metformin treatment vs. other oral hypoglycemic agents, and showed that diabetes adversely affects stroke prognosis. We also show that in diabetic patients with acute stroke, metformin-treatment was associated with a better outcome.

Interestingly, there is also a single report from Europe outlining the chronic use of metformin and prognosis (25). When compared to sulphonylureas treatment, patients treated with metformin had a better prognosis. Hematoma location or evacuation did not modify the association between metformin and mortality (25). Another more recent report from China however was not able to confirm these findings in a larger cohort of patients with intracerebral hemorrhage (26). The difference in outcome may be related to the small number of patients in the studies, the ethnicity, and types of hemorrhages in the two populations.

Understanding the neuroprotective mechanisms of metformin has been a subjective of recent reviews (4, 27–29). These effects include its ability to improve the balance of survival and death signaling in multiple cell types, including neurons, by improving energy metabolism, and reducing oxidative stress and proteostasis (3). These effects, especially the antioxidant effects, may be related to activation of the AMPK, an enzyme with important regulatory functions in energy regulation at multiple organs including the brain cells. AMPK is highly expressed in neuronal cells and is a major sensor of energy balance, especially in ischemic conditions (4, 5, 27–29). Metformin may also provide protective effects on the integrity of the neurovascular unit by improving function of endothelial cells, pericytes, astrocytes, and the blood brain barrier integrity (6). Additional effects include inhibition of inflammatory responses and prevention of the breakdown of the blood–brain barrier (8). These effects require chronic use of the medication to be effective as is evident from the research in models of cerebral ischemia in animals.

We have recently shown that upregulation of AMPK upregulation may be an important mechanism in preconditioning-induced neuroprotection in focal ischemia in rodents (30). Metformin, by increasing sublethal lactate levels in the brain, may also have similar preconditioning effects to explain neuroprotection (31). This effect is evident if the metformin is administered chronically. Acute administration of metformin immediately prior to focal ischemia may result in higher levels of lactate and paradoxically more severe stroke. This effect was dependent on the presence of AMPK and endothelial nitrous oxide (eNOS). The neuroprotective effects were not evident in AMPK and eNOS knockout mice (31). In another series of experiments with prolonged follow-up post-induction of focal ischemia in mice, the chronic use of metformin was associated with improved angiogenesis which likely contributed to the better outcome (32).

There is considerable amount of work on the protective effects of metformin in the cardiovascular literature. In patients with type 2 diabetes, metformin has been shown to reduce the incidence of cardiovascular disease and all-cause mortality (33). Similarly, metformin use has also been shown to reduce cardiovascular disease in individuals with prediabetes (34). A meta-analysis of studies related to the use of metformin in cardiovascular disease confirms these findings (35). Taken together with our study and the previous studies showing protection in stroke, the effects of metformin appears to be global in atherosclerotic diseases and promises to be a first-line medication in patients with diabetes and prediabetes, especially individuals that are at high risk for diabetic vascular complications.

Our study has several strengths. This is a large cohort with nearly 50% of patients with a diagnosis of diabetes and the data were prospectively collected. The comparison between diabetic and nondiabetic patients showed an association in slower recovery and diabetes. There were also over 1,130 patients on metformin and the comparison with other hypoglycemic again confirms that metformin use improves recovery and reduces mortality over the 90-day follow-up. Another important strength of the study is that we included the patients in the entire spectrum of symptoms and not only the patients that were candidates for thrombolysis. The larger number of patients with milder symptoms reflects the higher number of patients with subcortical lacunar strokes that are seen in increasing frequency in patients with hypertension and diabetes.

There are some limitations in our study. The main limitation of the study is that this was not a randomized or prospective study. It confirms similar observations in a smaller study from Japan (10) and in a multicenter study in patients being treated with intravenous rt-PA (15). We did not accurately document the duration of diabetes prior to the acute symptoms. The low incidence of long-term diabetes-related complications in our cohort suggests that very few patients had the disease for a long time. Another limitation of the study is that we did not have prior duration of treatment with oral hypoglycemic agents, due to which it was difficult to relate any neuroprotective effect of duration of metformin use in our study. Also the lack of accurate documentation of adherence to oral hypoglycemic agent use prior to the stroke is also another important shortcoming of the study. Another possible limitation is that we were unable to determine if there were any drug-to-drug interactions between the additional oral hypoglycemic medications that the patients were taking prior to the stroke. Such interactions are however very difficult to determine when the data are analyzed in retrospective studies. We also lacked long-term follow-up in our study. The long-term effects of metformin on endothelial function and on recurrent stroke will be important to evaluate.

In conclusions, we report on a large number of ischemic stroke patients and show that patients with diabetes on chronic pre-stroke treatment with metformin have improved recovery following the ischemic event. Metformin has been shown to have neuroprotective effects in animal models of focal ischemia and have vascular protective effects. The better prognosis and lower mortality may be secondary to these effects.

## Data Availability Statement

The original contributions presented in the study are included in the article/supplementary material, further inquiries can be directed to the corresponding author.

## Ethics Statement

The studies involving human participants were reviewed and approved by the Institutional Review Board of Hamad Medical Corporation. The patients/participants provided their written informed consent to participate in this study.

## Author Contributions

NA and AS: concept, design, and draft. NA, SJ, DM, BB, and SS: acquisition, analysis, interpretation of data, and technical and administrative support. SK: critical review. RS: statistical analysis. All authors contributed to the article and approved the submitted version.

## Conflict of Interest

NA, RS, SK, BB, SS, SJ, and DM were employed by Hamad Medical Corporation. The remaining author declares that the research was conducted in the absence of any commercial or financial relationships that could be construed as a potential conflict of interest.

## Publisher's Note

All claims expressed in this article are solely those of the authors and do not necessarily represent those of their affiliated organizations, or those of the publisher, the editors and the reviewers. Any product that may be evaluated in this article, or claim that may be made by its manufacturer, is not guaranteed or endorsed by the publisher.
